# A single intranasal dose of human mesenchymal stem cell-derived extracellular vesicles after traumatic brain injury eases neurogenesis decline, synapse loss, and BDNF-ERK-CREB signaling

**DOI:** 10.3389/fnmol.2023.1185883

**Published:** 2023-05-22

**Authors:** Maheedhar Kodali, Leelavathi N. Madhu, Roxanne L. Reger, Bojana Milutinovic, Raghavendra Upadhya, Sahithi Attaluri, Bing Shuai, Goutham Shankar, Ashok K. Shetty

**Affiliations:** Institute for Regenerative Medicine, Department of Cell Biology and Genetics, Texas A&M University School of Medicine, College Station, TX, United States

**Keywords:** cyclic AMP response-element binding protein, extracellular vesicles, hippocampal neurogenesis, mesenchymal stem cells, synapse loss, traumatic brain injury, brain-derived neurotrophic factor

## Abstract

An optimal intranasal (IN) dose of human mesenchymal stem cell-derived extracellular vesicles (hMSC-EVs), 90 min post-traumatic brain injury (TBI), has been reported to prevent the evolution of acute neuroinflammation into chronic neuroinflammation resulting in the alleviation of long-term cognitive and mood impairments. Since hippocampal neurogenesis decline and synapse loss contribute to TBI-induced long-term cognitive and mood dysfunction, this study investigated whether hMSC-EV treatment after TBI can prevent hippocampal neurogenesis decline and synapse loss in the chronic phase of TBI. C57BL6 mice undergoing unilateral controlled cortical impact injury (CCI) received a single IN administration of different doses of EVs or the vehicle at 90 min post-TBI. Quantifying neurogenesis in the subgranular zone-granule cell layer (SGZ-GCL) through 5′-bromodeoxyuridine and neuron-specific nuclear antigen double labeling at ~2 months post-TBI revealed decreased neurogenesis in TBI mice receiving vehicle. However, in TBI mice receiving EVs (12.8 and 25.6 × 10^9^ EVs), the extent of neurogenesis was matched to naive control levels. A similar trend of decreased neurogenesis was seen when doublecortin-positive newly generated neurons were quantified in the SGZ-GCL at ~3 months post-TBI. The above doses of EVs treatment after TBI also reduced the loss of pre-and post-synaptic marker proteins in the hippocampus and the somatosensory cortex. Moreover, at 48 h post-treatment, brain-derived neurotrophic factor (BDNF), phosphorylated extracellular signal-regulated kinase 1/2 (p-ERK1/2), and phosphorylated cyclic AMP response-element binding protein (p-CREB) levels were downregulated in TBI mice receiving the vehicle but were closer to naïve control levels in TBI mice receiving above doses of hMSC-EVs. Notably, improved BDNF concentration observed in TBI mice receiving hMSC-EVs in the acute phase was sustained in the chronic phase of TBI. Thus, a single IN dose of hMSC-EVs at 90 min post-TBI can ease TBI-induced declines in the BDNF-ERK-CREB signaling, hippocampal neurogenesis, and synapses.

## Introduction

The addition of new neurons occurs throughout life in the dentate gyrus of the hippocampus, a brain region vital for learning, memory, and mood function (Cameron and McKay, 1999; van Praag et al., 2002; Ming and Song, 2005). Such adult neurogenesis has been observed in various mammalian species, including humans, although there is no consensus on the extent of hippocampal neurogenesis in the adult human brain (Boldrini et al., 2018; Sorrells et al., 2018; Moreno-Jiménez et al., 2019). The contribution of hippocampal neurogenesis to learning and memory, spatial memory retention, contextual fear memory, and cognitive flexibility has been well recognized (Kheirbek et al., 2012; Besnard and Sahay, 2016; Terranova et al., 2019). Notably, the function of the dentate gyrus, including the maintenance of hippocampal neurogenesis, is considered necessary for pattern separation, an ability to transform similar inputs into distinctive representations, which helps distinguish highly similar environments (Aimone et al., 2011; Redell et al., 2020). The newly born neurons (i.e., immature granule cells) in the subgranular zone-granule cell layer (SGZ-GCL) of the dentate gyrus are purported to play a vital role in this process. Specifically, immature granule cells contribute to pattern separation by altering the activity of inhibitory interneurons and the response of mature granule cells to specific stimuli (Aimone et al., 2011; Redell et al., 2020).

Brain insults such as traumatic brain injury (TBI) can substantially alter hippocampal neurogenesis in acute and chronic phases. In the acute phase of TBI, enhanced neurogenesis occurs with increased proliferation of neural stem cells (NSCs), which may be the response of NSCs to promote innate cognitive recovery after brain injury (Chirumamilla et al., 2002; Gao and Chen, 2009). The TBI-induced increased neurogenesis is apparent 24 h after a controlled cortical impact injury (CCI), which continues for 7 days with a peak around 72 h post-injury. The extent of neurogenesis returns to baseline at 2 weeks post-TBI (Dash et al., 2001; Yi et al., 2013; Clark et al., 2020). TBI can also transiently alter the synaptic plasticity of newly born granule cells (Weston et al., 2021). Studies on functional implications of altered neurogenesis in TBI models have suggested that increased neurogenesis post-TBI can improve neurological outcomes (Kleindienst et al., 2005; Sun et al., 2010; Xiong et al., 2011). Also, disruption of neurogenesis post-TBI can exacerbate hippocampus-dependent learning and memory dysfunction (Blaiss et al., 2011; Sun et al., 2015). Nonetheless, some studies have also implied detrimental effects of increased neurogenesis in the acute phase of TBI, partly due to aberrantly migrated newly born neurons establishing inappropriate synaptic connections leading to increased seizure susceptibility (Villasana et al., 2015; Ibrahim et al., 2016; Neuberger et al., 2017).

Only a few studies have examined changes in hippocampal neurogenesis in the chronic phase of TBI. A study employing a lateral fluid percussion injury model reported comparable neurogenesis between sham and TBI groups at 31 days post-TBI (Clark et al., 2020). However, several studies using CCI models reported a substantial decline in neurogenesis at 28–35 days post-TBI (Wu et al., 2018; Shahror et al., 2020). A study quantifying neurogenesis at 70 days post-CCI reported a ~ 80% decline in neurogenesis (Watanabe et al., 2013). Thus, in the CCI model, the chronic phase of TBI is associated with a substantial decrease in neurogenesis. Such decline in the chronic phase of TBI is associated with impairments in spatial reference and working memories and pattern separation (Watanabe et al., 2013; Kim et al., 2016; Kodali et al., 2023). The precise mechanisms underlying decreased neurogenesis in the chronic phase of TBI are unknown. However, it is widely believed that the neuroinflammatory environment in the chronic phase of TBI is likely one of the contributors to diminishing neurogenesis. A previous study in a model of CCI has revealed the activation of nucleotide-binding domain, leucine-rich–containing family, pyrin domain–containing-3 (NLRP3) inflammasomes in the acute phase of TBI. Such activation continues into the chronic period of TBI leading to hyperactivation of p38 mitogen activated protein kinase (p38/MAPK) signaling and continuous release of proinflammatory cytokines, which maintains a chronic neuroinflammatory state in the hippocampus and the injured cerebral cortex (Kodali et al., 2023). The study also showed that intranasal (IN) dispensation of human mesenchymal stem cell-derived extracellular vesicles (hMSC-EVs), inherently comprising microglia-modulating miRNAs, can suppress NLRP3 inflammasome activation within microglia in the acute phase. Interestingly, such suppression was endured in the chronic phase, preventing the hyperactivation of p38 MAPK signaling and perpetuation of the neuroinflammatory environment in the hippocampus (Kodali et al., 2023).

Chronic neuroinflammation, declined neurogenesis, and synapse loss can contribute to TBI-induced enduring cognitive and mood dysfunction. Accordingly, the present study investigated whether hMSC-EV treatment after TBI can prevent hippocampal neurogenesis decline and synapse loss in the chronic phase of TBI. Furthermore, chronic neuroinflammation mediated by microglia likely impacts neurogenesis and synapse loss. Hence, it is interesting to investigate the effects of hMSC-EV treatment-linked modulation of proinflammatory microglia into noninflammatory microglia on neurogenesis and synapse loss in a TBI model (Kodali et al., 2023). The concept of using hMSC-EVs for treating TBI is based on their antiinflammatory and neuroprotective properties observed in previous studies in models of status-epilepticus and TBI (Kim et al., 2016; Long et al., 2017). Furthermore, to understand the potential mechanisms by which hMSC-EVs could reduce the hippocampal neurogenesis decline, brain-derived neurotrophic factor (BDNF), phosphorylated extracellular signal-regulated kinase 1/2 (p-ERK1/2), and phosphorylated cyclic AMP response-element binding protein (p-CREB) levels were measured in the injured hippocampus after TBI with vehicle or hMSC-EV treatment.

## Materials and methods

### Animals and study design

**Figure 1:**
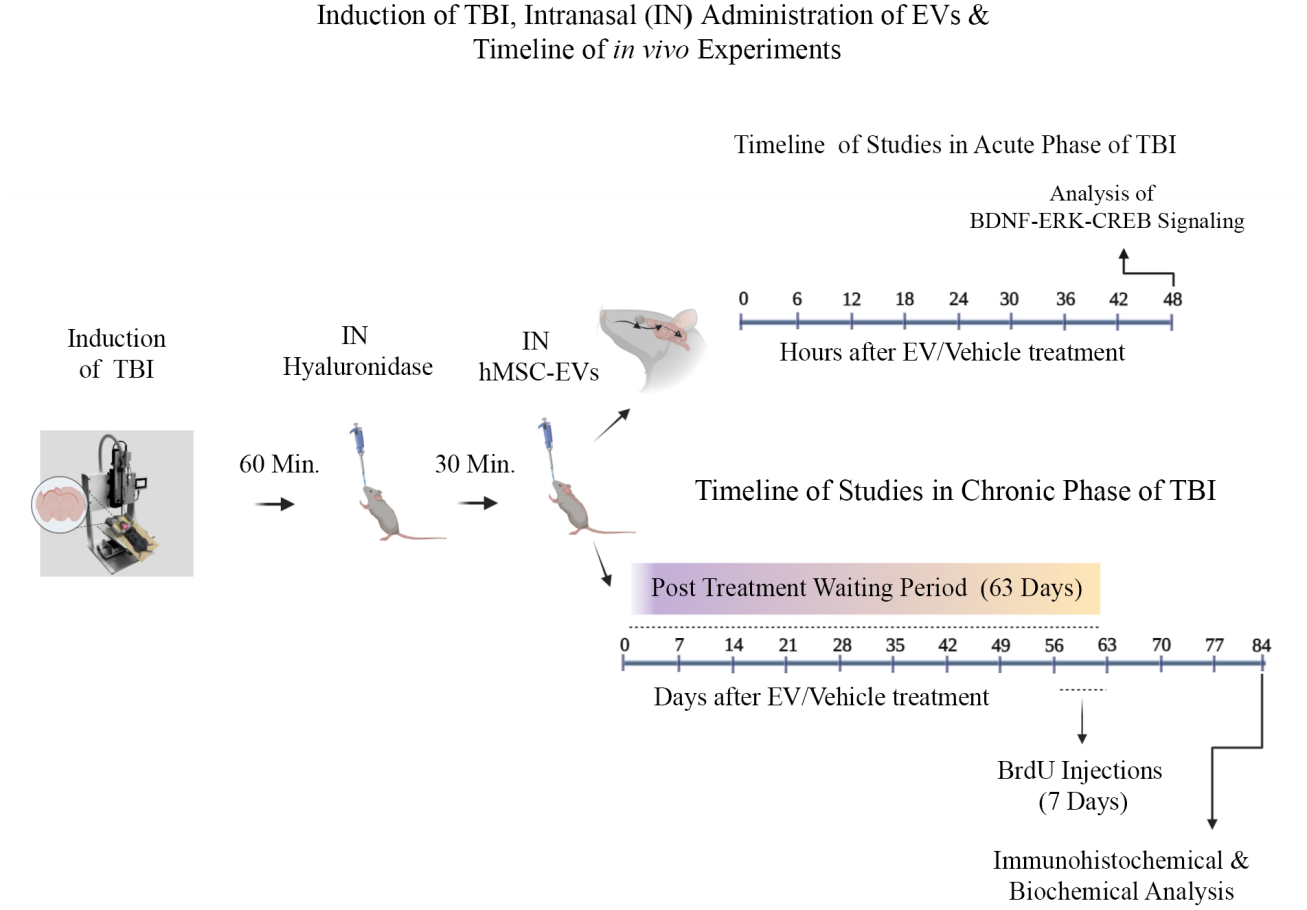
A graphic representation of different experiments conducted in the study. These include a controlled cortical impact injury induction, intranasal (IN) hyaluronidase treatment at 60 min post-TBI, IN administration of hMSC-EVs at 90 min after TBI, and the sequence of investigations in the acute and chronic periods of TBI. The studies in the acute phase of TBI comprise characterization of brain-derived neurotrophic factor (BDNF), phosphorylated extracellular signal-regulated kinase 1/2 (p-ERK1/2), and phosphorylated cAMP-response element binding protein (p-CREB) in the injured brain at 48 h after vehicle or hMSC-EV treatment. The studies in the chronic phase of TBI involve 5′-bromodeoxyuridine (BrdU) injections for 7 days at 56–63  days after TBI and brain tissue collection at 84  days post-injury and hMSC-EV treatment.

Two-month-old male C57BL/6J mice procured from Jackson Laboratories (Bar Harbor, ME, USA) were utilized. The Animal Care and Use Committee of Texas A&M University approved all procedures performed on animals. A schematic diagram showing the timelines of experiments and analyses performed in this study is illustrated in Figure 1. Animals were randomly assigned to naïve control, sham surgery, and TBI groups (*n* = 10/group). Ninety minutes after the induction of controlled cortical impact injury (CCI), animals in TBI groups were treated with an IN dose of vehicle (phosphate buffered saline [PBS]; referred to as TBI) or hMSC-EVs (6.4, 12.8, or 25.6 × 10^9^ EVs/mice) (Kodali et al., 2023). A subgroup of animals in every group (*n* = 6/group) received intraperitoneal injections of 5′-bromodeoxyuridine (BrdU, 100 mg/Kg/day for 7 days) at post-TBI days 57–63 after sham or TBI surgery for neurogenesis analysis. Age-matched subgroups of animals in the naïve control and sham surgery groups neither received vehicle nor EVs. However, they were subjected to similar BrdU injections at time points matching the TBI groups. Eighty-four days post-TBI (equivalent to 3 weeks post-BrdU injections), animals receiving BrdU in all groups were perfused intracardially with 4% paraformaldehyde and brain tissues were processed for immunohistochemical and immunofluorescence studies. Fresh brain tissues were harvested from a second subgroup of animals in every group (*n* = 4/group) for biochemical assays. The age of the mice at the time of euthanasia was ~5 months in these long-term studies. Furthermore, to investigate changes in the concentration of mature BDNF, p-ERK1/2, and p-CREB in the hippocampus ipsilateral to CCI, additional subgroups of naïve control mice (*n* = 6), TBI mice receiving the vehicle (*n* = 6), and TBI mice receiving 25.6 × 10^9^ EVs (*n* = 6) were euthanized at 48 h post-treatment, and fresh brain tissues were harvested. The various treatment groups and the number of mice employed per group for different data collection and analyses are presented in Table 1. The animal groups and treatments were blinded to investigators who collected and analyzed data.

**Table 1:** Treatment groups and the number of mice employed per group for different analyses.

Groups	Number of mice employed/group in different analyses				
	BrdU labeling studies	DCX+ neuronal counts	Syn + and PSD95+ puncta measurements	Biochemical Assays - acute phase of TBI	Biochemical Assays - chronic phase of TBI
Naïve	6	6	5–6	6	4–6
Sham	6	6	–	–	–
TBI	6	6	5–6	6	4–6
TBI + EV6.4	6	5	5–6	–	–
TBI + EV12.8	5	6	5–6	–	–
TBI + EV25.6	6	6	5–6	6	4–6

BrdU, 5′-bromodeoxyuridine, DCX, doublecortin; PSD95, postsynaptic density protein 95; Syn, synaptophysin; TBI, traumatic brain injury; EV6.4, 6.4 × 10^9^ extracellular vesicles (EVs); EV12.8, 12.8 × 10^9^ EVs; EV25.6, 25.6 × 10^9^ EVs.

### Induction of controlled cortical impact injury (CCI)

A unilateral CCI was induced in animals belonging to TBI and TBI + hMSC-EV groups using a CCI device (Leica Biosystems, Deer Park, IL, USA) attached to stereotaxic equipment (MyNeurolab, Richmond, IL, USA). Mice in the sham surgery group received craniotomy but did not receive CCI, whereas mice in the naive control group received neither craniotomy nor hMSC-EVs treatment. For craniotomy, the mice were anesthetized with intramuscular injection of a cocktail comprising ketamine (30 mg/Kg), acepromazine (0.25 mg/Kg), and xylazine (3 mg/Kg). For inducing CCI, following anesthesia, the head of the mouse was fixed to a stereotactic frame (Kodali et al., 2023). Craniotomy (measuring ~4 mm in diameter) was performed on the right half of the calvaria after a midline incision. The region of craniotomy included an area overlying the temporoparietal cortex, with the center located midway between bregma and lambda and 2 mm away from the midline. A single impact was delivered to each mouse with a 3 mm diameter impactor tip, which penetrated 0.8 mm into the brain at a speed of 5 m/s and stayed for 300 milliseconds. Using tissue adhesive, a disk made from dental cement was placed on the craniotomy area, and surgical skin clips were used to close the skin. The animal was maintained in a temperature-controlled cage and monitored. The mice in the TBI group received IN administration of vehicle (PBS), whereas mice in the TBI + hMSC-EV groups received IN administration of 6.4, 12.8, or 25.6 × 10^9^ hMSC-EVs in PBS. To alleviate pain, all animals undergoing survival surgery received buprenorphine injections (0.1 mg/Kg, twice daily on days 1–2 and once daily on days 3–4). In addition, the mice were given 1 mL subcutaneous saline injections daily for 4 days to prevent dehydration.

### Expansion of hMSC cultures, and isolation of hMSC-EVs for IN administration

The protocols employed for the culture and expansion of human bone marrow-derived hMSCs, and purification and characterization of hMSC-EVs, are described in the previous publications (Kim et al., 2016; Long et al., 2017; Kodali et al., 2023). Briefly, passage 4 (P4) hMSCs were cultured in a complete culture medium (CCM) and fed every 2 days. The CCM comprised alpha minimal essential medium (Invitrogen, Waltham, MA, USA), fetal bovine serum (Atlantic Biologicals, Miami, FL, USA), L-glutamine or glutamax (Invitrogen), and penicillin/streptomycin (Invitrogen). After ~70% confluence, the CCM was replaced with a commercially available serum-free medium (CD-CHO medium, Invitrogen) optimized with supplements. Six hours later, the medium was replaced with a fresh CD-CHO medium. After 48 h, the media from hMSC cultures were collected for the isolation of EVs. Harvested culture media were centrifuged to remove debris, filtered through 0.22 μm Stericups, aliquoted, and stored at −20^o^C. After thawing, EVs in the media were isolated by anion-exchange chromatography (AEC), as detailed in the previously published reports (Kim et al., 2016; Kodali et al., 2023). NanoSight, a device used to measure the nano-sized particles (Malvern Panalytical, Malvern, UK), was used to measure the concentration and the size of EVs. The characterization of EVs for EV-specific marker expression using western blots and enzyme-linked immunoassays, ultrastructure using transmission electron microscopy, and antiinflammatory activity using lipopolysaccharide-stimulated mouse macrophage cultures are detailed in the previous studies (Kodali et al., 2023). The hMSC-EVs were intranasally dispensed bilaterally, as detailed in the previous publications (Kodali et al., 2019; Upadhya et al., 2020). Briefly, 1 h after TBI induction, each nostril was treated with hyaluronidase (100 U, Sigma-Aldrich, St. Louis, MO, USA) to enhance the permeability of the nasal mucous membrane. Thirty minutes later, the prep comprising hMSC-EVs was administered bilaterally via the IN route (5 μL/nostril each time).

### Tissue processing and immunohistochemistry

The methods for brain tissue processing are similar to that described in the previous reports (Shetty et al., 2017; Kodali et al., 2018). In brief, animals were deeply anesthetized, perfused intracardially with 4% paraformaldehyde, and the brains removed. The brains were post-fixed overnight in 4% paraformaldehyde and processed for cryostat sectioning following cryoprotection with different concentrations of sucrose. The entire forebrain was sectioned coronally at thirty-micrometer thickness, and serial sections were collected and stored in an anti-freezing solution at −30 degrees centigrade. Every 15th section through the entire septo-temporal axis of the hippocampus was processed for BrdU or doublecortin (DCX) immunohistochemistry using avidin-biotin complex methods, as described in the previous reports (Hattiangady et al., 2008; Shetty et al., 2011). Five serial sections (every 15th) covered sampling from the entire hippocampus. The primary antibodies for visualizing BrdU+ newly generated cells and DCX+ immature neurons comprised a rat anti-BrdU (1:200 Abcam) and a goat anti-DCX (1:300, Abcam, Cambridge, MA, USA). The secondary antibodies comprised biotinylated anti-goat or anti-rat IgGs (1:250, Vector Labs, Burlingame, CA, USA). The study employed diaminobenzidine (Vector Labs) or vector SG (Vector Labs) as chromogens. After washing, the sections were mounted on 0.5% gelatin-coated slides, as described in the previous study.

### Dual immunofluorescence methods

Dual immunofluorescence procedures were employed to visualize BrdU+ cells expressing neuron-specific nuclear protein (NeuN) and pre-and post-synaptic proteins positive for synaptophysin (Syn) and post-synaptic density protein 95 (PSD95). These studies utilized three representative sections, one each from the anterior, mid, and posterior levels of the hippocampus. The detailed methods are available in the previous reports (Hattiangady and Shetty, 2010; Attaluri et al., 2022). The sections were washed in phosphate buffer saline (PBS), blocked with 10% normal donkey serum, and incubated overnight at 4°C in a cocktail of two primary antibodies (anti-rabbit NeuN and anti-rat BrdU or anti-rabbit Syn and anti-goat PSD95). The following day, the sections were treated with the appropriate secondary antibodies, rinsed in PBS, and coverslipped with a slow fade/antifade mounting medium (Invitrogen). The primary antibodies utilized were anti-rabbit NeuN (1:1000, Millipore Sigma, St. Louis, MO, USA), anti-rat BrdU (1:100, Abcam), anti-rabbit Syn (1:500, synaptic systems, Gottingen, Germany), and anti-goat PSD95 (1:500, Abcam). The secondary antibodies comprised donkey anti-rat IgG conjugated with Alexa Flour 594 (1:200, Invitrogen, Grand Island, NY, USA), donkey anti-rabbit IgG conjugated with Alexa Flour 488 (1:200, Invitrogen), donkey anti-goat IgG conjugated with Alexa Flour 594 (1:200, Invitrogen).

### Quantification of BrdU+ newly born cells and DCX+ newly born neurons

Every 15th thirty-micrometer section through the entire hippocampus was used for quantifying the numbers of BrdU+ newly born cells or DCX-positive newly born neurons in the SGZ-GCL of the hippocampus (*n* = 5 sections/marker/animal). A StereoInvestigator system (Microbrightfield, Williston, Vermont, USA) was employed to quantify BrdU+ and DCX+ cells using 100X objective lens and stereological methods, as detailed in the previous reports (Hattiangady et al., 2007; Kodali et al., 2021). The numbers of BrdU+ cells and DCX+ cells quantified using StereoInvestigator represent absolute counts for the entire SGZ-GCL of the hippocampus.

### Measurement of neuronal differentiation of BrdU+ newly born cells, and quantification of net hippocampal neurogenesis

For quantifying the percentage of BrdU+ cells displaying NeuN expression in the SGZ-GCL, we employed Z-section analysis using a 40X objective lens in a Nikon confocal microscope. These studies utilized three representative sections, one each from the anterior, mid, and posterior levels of the hippocampus processed for BrdU-NeuN dual immunofluorescence (Shetty et al., 2012, 2020). In every animal, 25–30 BrdU+ cells were evaluated from 3 representative sections for NeuN expression (*n* = 5–6/group). Next, the numbers of new neurons born at post-TBI days 57–63 and survived for 3 weeks were determined for the hippocampus of TBI mice receiving vehicle or hMSC-EVs, age-matched naïve mice, and mice undergoing sham TBI via respective absolute counts of BrdU+ cells determined through stereology and percentages of BrdU+ cells expressing NeuN in the SGZ-GCL determined through Z-section analysis in a confocal microscope (Shetty et al., 2012, 2020). Thus, extrapolating the absolute numbers of BrdU+ cells with the respective percentages of BrdU+ cells expressing NeuN facilitated quantifying the total number of mature neurons added to the GCL over 7 days in various groups.

### Measurement of pre- and post-synaptic proteins

Z-sections obtained from three representative brain tissue sections stained for Syn and PSD95 dual immunofluorescence in a Nikon confocal microscope, and ImageJ, were employed to quantify area fractions of Syn and PSD95 in the somatosensory cortex (SSC) and the dentate molecular layer (Kodali et al., 2021). The images comprising Syn+ (green) or PSD95+ (red) puncta were first converted into an 8-bit grayscale image using Image J. Then, after thresholding, for each image comprising Syn+ or PSD95+ puncta, the area fractions of Syn+ or PSD95+ structures were measured. In addition, we also quantified putative synapses (i.e., the contact sites between Syn+ and PSD95+ structures) from randomly selected regions each measuring 2.15 μm^2^. For each brain region, six Z-images collected from 3 representative sections from each animal (*n* = 5–6/group).

### Quantification of mature BDNF, p-ERK, and p-CREB from hippocampal tissue lysates

The frozen fresh brain samples were thawed, and the entire injured hippocampus (i.e., hippocampus ipsilateral to the CCI) from TBI mice receiving vehicle or hMSC-EVs was micro-dissected and placed in a tissue extraction reagent (Invitrogen, Waltham, MA) containing protease inhibitor (Sigma Aldrich, 1:100 dilution). The entire hippocampi from naïve controls were also micro-dissected and treated similarly. Each hippocampus was individually lysed using sonication for 15–20 s at 4°C. The resultant solution was centrifuged at 15,000 *g* for 10 min. The supernatant was then collected, aliquoted, and stored at −80°C for later use. Using kits, the concentrations of mature BDNF (R&D Systems, Minneapolis, MN, USA), p-ERK1/2, and p-CREB (Cell Signaling, Danvers, MA, USA) were measured from hippocampal lysates. In brief, hippocampal lysates were added to precoated 96-well plates with capture antibodies and incubated. After a thorough rinse in a wash buffer, specific biotin-conjugated detection antibodies were added and incubated at room temperature. Following the wash, the plates were incubated with streptavidin-HRP conjugate with gentle shaking. The plates were then treated with substrate solution, and the reaction was terminated by adding a stop solution before being measured at 450 nm. The concentration of each protein was determined using standard graphs. All protein values were normalized to 1 mg of total protein in the tissue lysate.

### Western blots for Syn and PSD-95

After determining the total protein concentration from SSC tissue lysates (*n* = 6/group) by BCA protein assay kit (ThermoFisher Scientific, Waltham, MA, USA), 30 μg of protein were loaded onto 4–12% NuPAGE Bis-Tris Gels (ThermoFisher Scientific) and separated. After transferring proteins onto a nitrocellulose membrane using the iBlot2 gel transfer device (ThermoFisher Scientific), proteins in the membrane were detected using antibodies against Syn (1:5000, ProteinTech, Rosemont, IL, USA), PSD95 (1:1000, Abcam) and GAPDH (1:1000, Millipore). GAPDH was used as a loading control for cortex tissue lysates. Then, the protein signals were detected using the chemiluminescence reagents provided with the ECL detection kit (ThermoFisher Scientific) and visualized using the iBright imaging system (ThermoFisher Scientific). ImageJ software was used to determine the intensity of each targeted band (Syn, 38 kDa; PSD95: 95 kDa) by normalizing it to a corresponding GAPDH band, also run along with Syn and PSD95.

### Statistical analyses

Power analysis using G*Power software suggested that for the effect size (delta) of 0.93 and an alpha of 0.05, 4 mice/group are sufficient to obtain a power of over 0.8. Therefore, we obtained final data from 4 to 6 mice in all assays in the study. One-way analysis of variance (ANOVA) with Tukey’s multiple comparison *post hoc* tests was employed for detecting differences between three or more datasets. Furthermore, we conducted the non-parametric Kruskal-Wallis test, followed by Dunn’s *post hoc* tests when individual groups did not pass the normality test (Shapiro–Wilk test). *p* < 0.05 was taken as a statistically significant value in all measurements.

## Results

### hMSC-EV treatment after TBI preserved hippocampal neurogenesis

**Figure 2:**
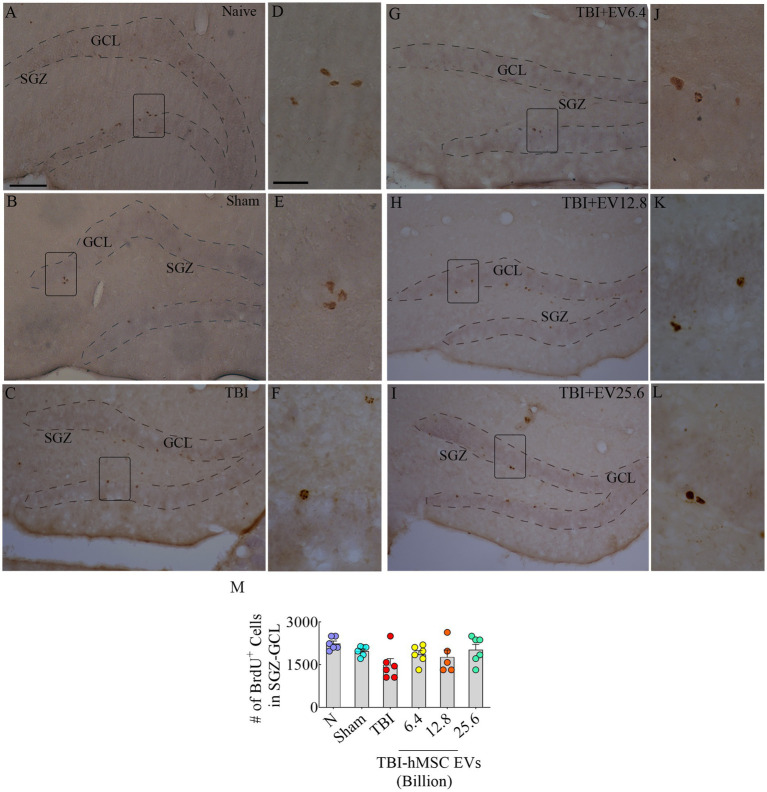
5’bromodeoxyuridine (BrdU)-labeled newly born cells from different groups in the subgranular zone-granule cell layer (SGZ-GCL). **(A–L)** Illustrates examples of BrdU+ newly born cells from naïve, sham, TBI **(A–C)**, and TBI + EV (6.4–25.6 × 10^9^, **G–I**) groups. **(D–F,J–L)** Illustrate the magnified images of the respective groups. Bar chart **(M)** compares the number of BrdU+ newly born cells in the SGZ-GCL of the hippocampus between different groups. The scale bar in **A** (100 μm) applies to **A–C** and **G–I**, and the scale bar in **D** (25 μm) applies to **D–F** and **J–L**.

**Figure 3:**
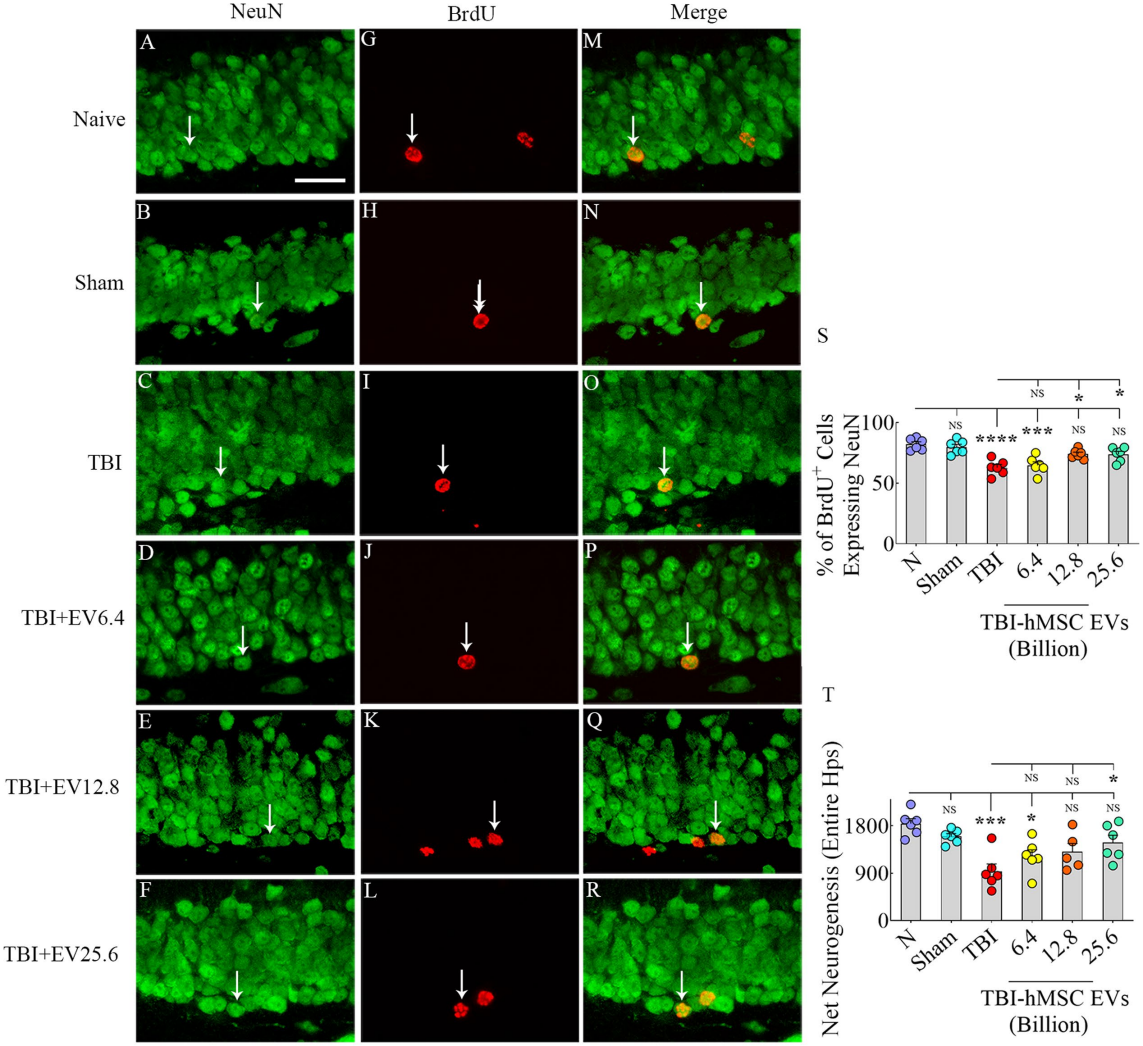
Intranasal hMSC-EV treatment in the acute phase of TBI eased hippocampal neurogenesis decline at 57–63 days post-TBI. **(A–R)** Illustrate examples of 5′-bromodeoxyuridine-positive (BrdU+) newly born cells that differentiated into NeuN+ neurons for naïve **(A,G,M)**, TBI **(B,H,N)** and TBI + EVs (6.4–25.6 × 10^9^, **C–R**), respectively. The bar charts **(S,T)** compare the percentage of BrdU+ cells that differentiated into NeuN+ neurons **(S)** and net hippocampal neurogenesis **(T)** in the SGZ-GCL of the hippocampus between different groups. Scale bar in **A** (25 μm) applies to **A–R**. *p < 0.05; ***p < 0.001; ****p < 0.0001; NS, not significant.

Through BrdU labeling and BrdU-NeuN dual immunofluorescence methods, we first evaluated the numbers of new neurons born in the injured hippocampus at post-TBI days 57–63 and survived for 3 weeks in TBI mice receiving vehicle or different doses of hMSC-EVs, in comparison to age-matched naïve control mice and sham TBI mice. Representative images showing the distribution of BrdU+ cells in the SGZ-GCL of different groups are illustrated in Figures 2A–C, G–I. Magnified images show the morphology of BrdU+ cells in different groups (Figures 2D–F, J–L). One-way ANOVA analysis of numbers of BrdU+ cells in the SGZ-GCL revealed no significant differences between naïve control mice, sham TBI mice, and TBI mice receiving vehicle or different doses of hMSC-EVs (*p* > 0.05, Figure 2M). Quantification of percentages of BrdU+ cells that expressed the mature neuronal marker NeuN in the SGZ-GCL enabled the quantification of net hippocampal neurogenesis. Representative images of BrdU+ cells differentiating into NeuN+ neurons in naïve, TBI, and TBI + EV groups (6.4–25.6 × 10^9^) are shown in Figures 3A–R. Notably, neuronal differentiation of newly generated cells diverged substantially between groups (*p* < 0.0001, Figure 3S). Compared to the naïve control group, the extent of neuronal differentiation was reduced in the TBI and TBI + 6.4 × 10^9^ EV group (*p* < 0.001–0.0001, Figure 3S). However, the neuronal differentiation of newly generated cells in TBI + 12.8 or 25.6 × 10^9^ EV groups was comparable to the naïve control group (*p* > 0.05, Figure 3S). Compared to the sham group also, the extent of neuronal differentiation was reduced in the TBI and TBI + 6.4 × 10^9^ EV group (*p* < 0.01–0.001, Figure 3S), but the degree of neuronal differentiation of newly generated cells in TBI + 12.8 or 25.6 × 10^9^ EV groups remained similar to the sham group (*p* > 0.05, Figure 3S).

Furthermore, the TBI mice that received higher doses of EVs (TBI + 12.8 or 25.6 × 10^9^) had significantly higher neuronal differentiation of newly generated cells than the TBI group (*p* < 0.05, Figure 3S). Because of the differences in neuronal differentiation, net hippocampal neurogenesis, computed by extrapolating the absolute number of BrdU+ cells for the entire SGZ-GCL (generated through stereological counts) with the percentage of BrdU+ cells that differentiated into NeuN+ neurons, also varied between groups (*p* < 0.001, Figure 3T). The net hippocampal neurogenesis was reduced in the TBI and TBI + 6.4 × 10^9^ EV groups compared to the naïve control group (*p* < 0.05–0.001, Figure 3T). However, the TBI + 12.8 or 25.6 × 10^9^ EV groups displayed comparable net neurogenesis as seen in the naïve control group (*p* > 0.05, Figure 3T). Compared to the sham group, net hippocampal neurogenesis was reduced in the TBI group (*p* < 0.01, Figure 3T), but net hippocampal neurogenesis in TBI + 6.4, 12.8 or 25.6 × 10^9^ EV groups remained similar to the sham group (*p* > 0.05, Figure 3T). Furthermore, the TBI + 25.6 × 10^9^ EV group displayed significantly better net neurogenesis than the TBI group (*p* < 0.05, Figure 3T). Thus, higher doses of hMSC-EV treatment in the acute phase of TBI preserved hippocampal neurogenesis at naïve control levels in the chronic phase of TBI.

### hMSC-EV treatment after TBI preserved hippocampal neurogenesis 84  days post-injury

**Figure 4:**
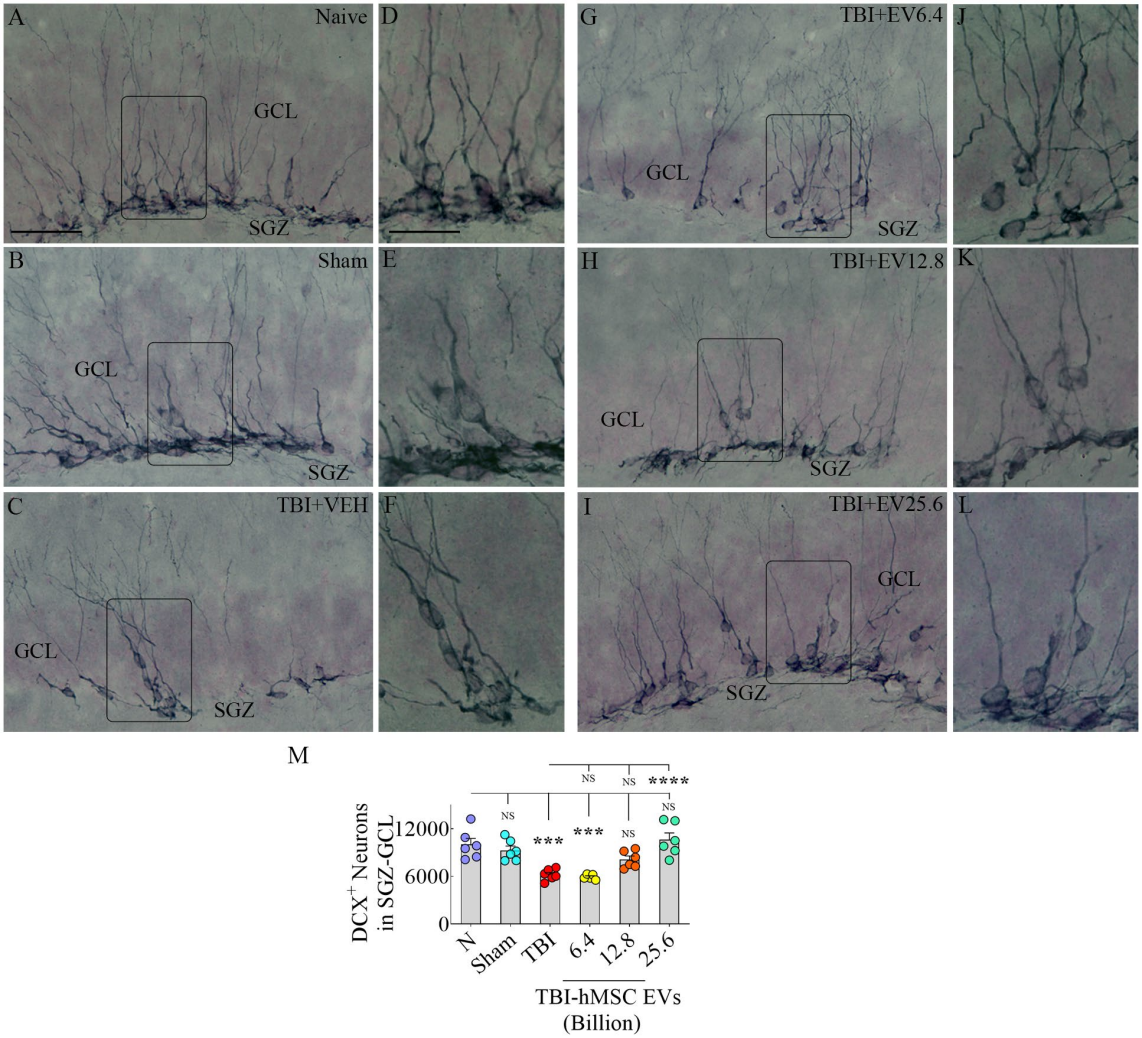
Intranasal hMSC-EV treatment in the acute phase of TBI eased hippocampal neurogenesis decline at 84 days post-TBI. **(A–L)** Show doublecortin positive (DCX+) newly born cells from naïve, sham, TBI **(A–C)**, and TBI + EVs (6.4–25.6 × 10^9^; **G–I**) groups. **(D–F,J–L)** Illustrate the magnified images of the respective groups. The bar chart **M** compares the number of DCX+ neurons in the subgranular zone-granule cell layer across groups. The scale bar in **A** (50 μm) applies to **A–C** and **G–I**, and the scale bar in **D** (25 μm) applies to **D–F** and **J–L**. ***p < 0.001; ****p < 0.0001; NS, not significant.

We next quantified DCX+ newly born neurons in the SGZ-GCL to assess the status of hippocampal neurogenesis at 84 days post-TBI. Representative images showing the distribution of DCX+ neurons in different groups are presented (Figures 4A–C, G–I). Magnified images revealed similar morphology of DCX+ neurons across groups (Figures 4D–F, J–L). Notably, signs of abnormal neurogenesis (i.e., the migration of newly born neurons into the dentate hilus or the dentate molecular layer were not seen in any of the groups). The dendrites of most neurons in all groups projected through the GCL into the inner and middle molecular layers of the dentate gyrus (Figures 4A–L). ANOVA analysis of the numbers of DCX+ neurons in the SGZ-GCL revealed significant differences between groups (*p* < 0.0001, Figure 4M). The DCX+ newly born neurons were reduced in the TBI and TBI + 6.4 × 10^9^ EV group compared to the naïve control group (*p* < 0.001, Figure 4M). In contrast, DCX+ neurons in the TBI + 12.8 or 25.6 × 10^9^ EV groups matched numbers in the naïve control group (*p* > 0.05, Figure 4M). The TBI + 12.8 or 25.6 × 10^9^ EV groups also displayed significantly higher DCX+ newly born neuron numbers than the TBI group (*p* < 0.05–0.0001, Figure 4M). Thus, higher doses of hMSC-EV treatment after TBI preserved the status of hippocampal neurogenesis at naïve control levels at 84 days post-TBI.

### hMSC-EV treatment after TBI prevented dysregulation of the BDNF-ERK-CREB signaling pathway

**Figure 5:**
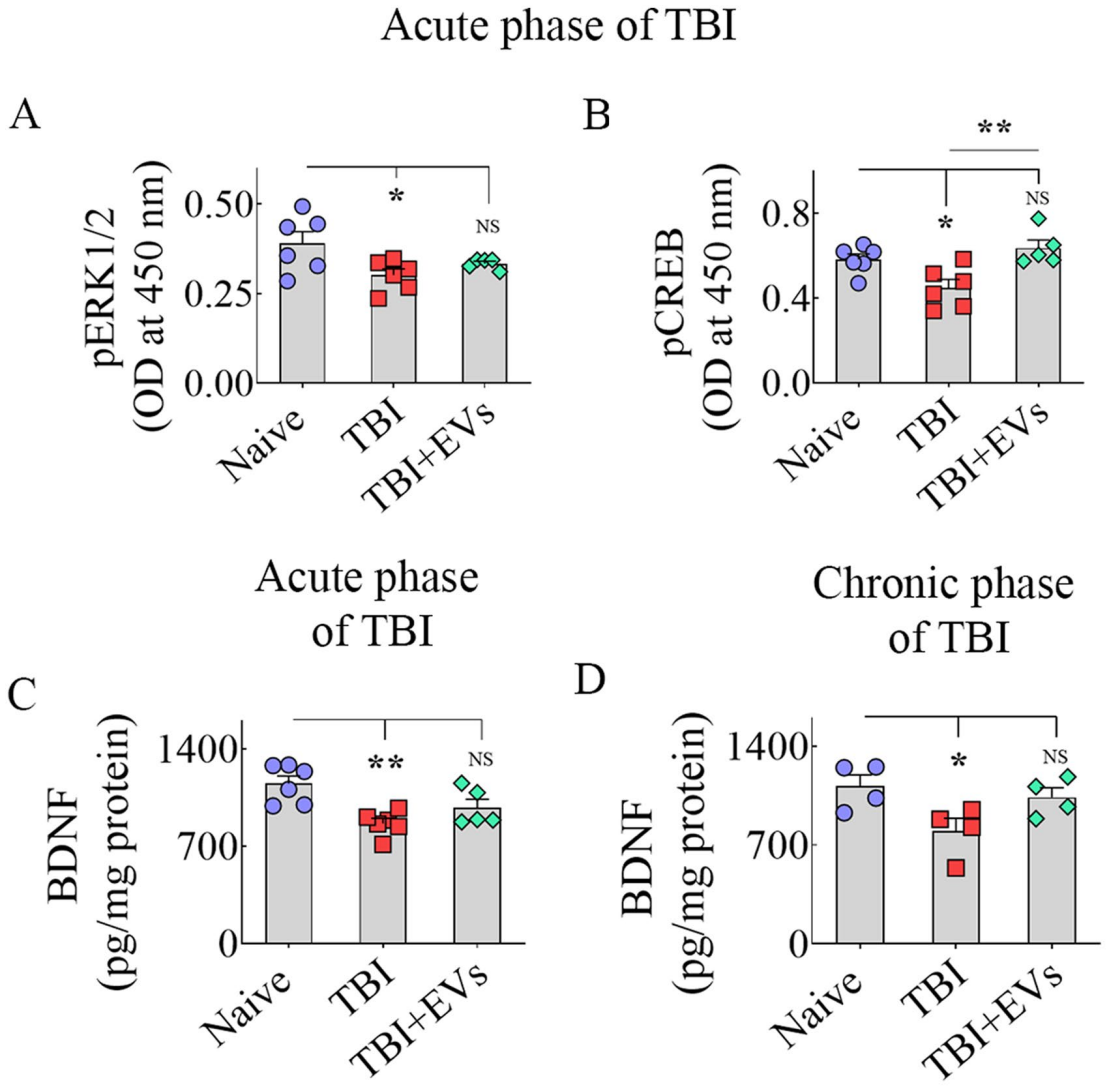
Intranasal hMSC-EV treatment after TBI improved BDNF-pERK-pCREB signaling. The bar charts **A**–**D** display the concentration of phosphorylated extracellular signal-regulated kinase (p-ERK, **A**), phosphorylated cAMP-response element binding protein (p-CREB, **B**), and brain-derived neurotrophic factor (BDNF, **C**) between naïve, TBI, and TBI + EVs (25.6 × 10^9^) groups at 48 h after TBI. The bar chart **(D)** compares the concentration of BDNF between naïve, TBI, and TBI + EV (25.6 × 10^9^) groups at 84 days post-EV treatment. *p < 0.05; **p < 0.01; NS, not significant.

Next, we investigated whether a single IN treatment of 25.6 × 10^9^ hMSC-EVs 90 min after TBI would ease the downregulation of the BDNF-ERK-CREB signaling pathway in the hippocampus ipsilateral to injury in the acute phase of TBI. We quantified the concentrations of major components of the BDNF-ERK-CREB signaling pathway, such as BDNF, pERK1/2, and *p*-CREB in the hippocampus, to grasp the potential mechanisms of hMSC-EV treatment-mediated maintenance of hippocampal neurogenesis at naïve control levels. ANOVA analysis uncovered differences between groups for pERK1/2, *p*-CREB, and BDNF levels (*p* < 0.05–0.01, Figures 5A–C). The TBI group exhibited a diminished concentration of pERK1/2 vis-à-vis the naïve control group (*p* < 0.05, Figure 5A). hMSC-EV treatment after TBI normalized pERK1/2 concentration to naïve control levels (*p* > 0.05, Figure 5A), but the EV treatment-mediated increase did not go significantly above the TBI group (*p* > 0.05, Figure 5A). A reduced *p*-CREB concentration was observed in the TBI group vis-à-vis the naïve control group (*p* < 0.05, Figure 5B). hMSC-EV treatment boosted the concentration of *p*-CREB. The overall increase did not statistically differ from the concentration in the naïve control group (*p* > 0.05, Figure 5B) but was enhanced compared to the TBI group (*p* < 0.01, Figure 5B). BDNF concentration was reduced in the TBI group compared to the naïve control group (*p* < 0.01, Figure 5C). hMSC-EV treatment increased the BDNF concentration closer to naïve control levels (*p* > 0.05, Figure 5C). However, the EV treatment-mediated augmentation did not rise significantly above the TBI group (*p* > 0.05, Figure 5C).

We also measured BDNF concentration in the hippocampus at 84 days post-EV treatment to address whether the increased concentration observed at 48 h post-EV treatment was sustained at 84 days post-EV treatment. This analysis showed that the hippocampus of TBI mice receiving EVs displayed BDNF concentration equivalent to the age-matched naïve control mice (*p* > 0.05, Figure 5D). In contrast, TBI mice receiving the vehicle displayed reduced BDNF concentration compared to naïve control mice (*p* < 0.05, Figure 5D). However, BDNF levels did not differ significantly between TBI and TBI + EVs groups (*p* > 0.05, Figure 5D). Overall, hMSC-EV treatment after TBI brought the concentrations of p-ERK1/2, *p*-CREB, and BDNF closer to naïve control levels in the acute phase of TBI. Furthermore, increased BDNF concentrations in TBI mice receiving EVs observed in the acute phase of TBI were sustained in the chronic phase of TBI. However, p-ERK1/2 and BDNF levels in the acute phase and BDNF concentration in the chronic phase did not differ significantly between TBI and TBI + EVs groups, suggesting a partial recovery of the BDNF-ERK-CREB signaling with hMSC-EV treatment.

### hMSC-EV treatment eased synapse loss after TBI

**Figure 6:**
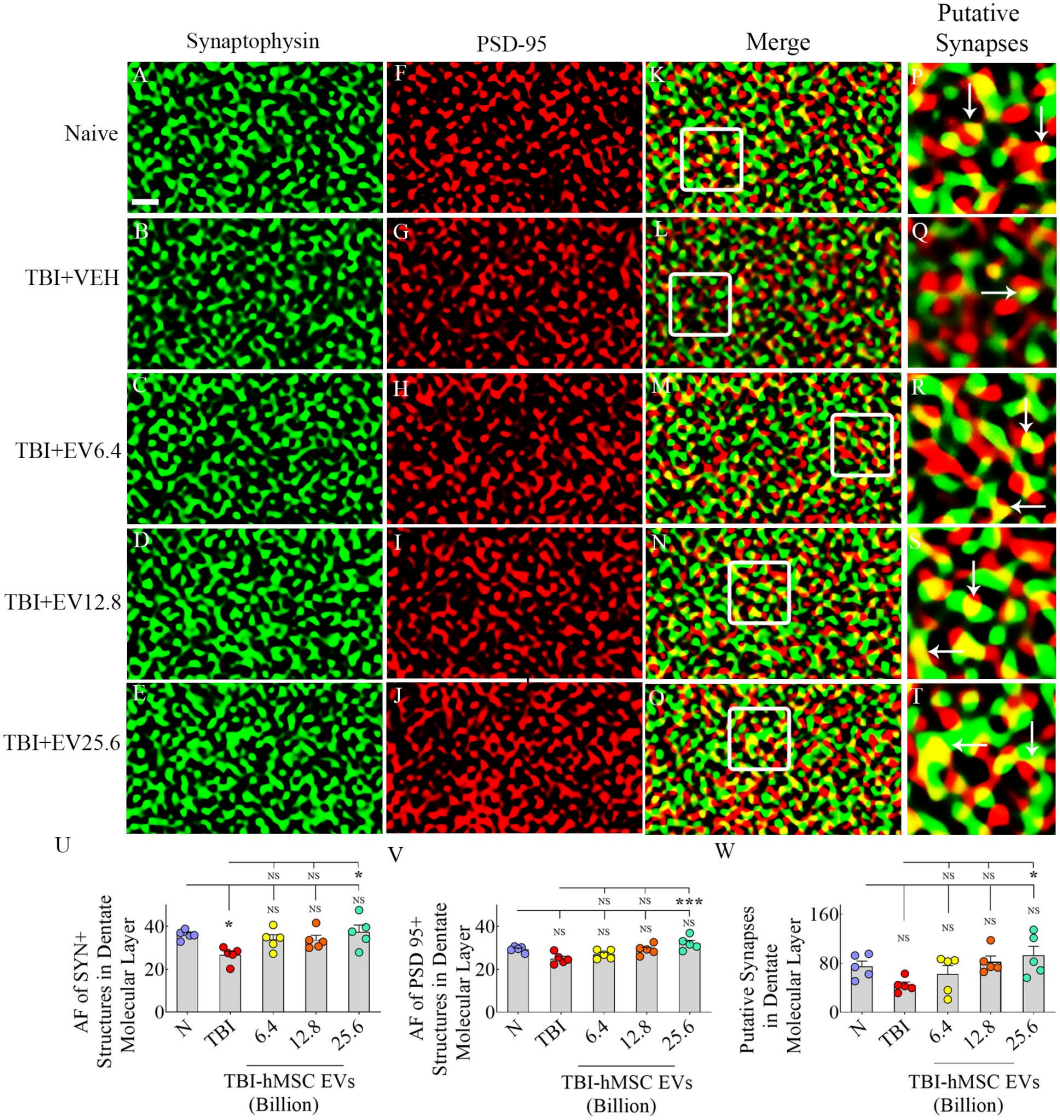
Intranasal hMSC-EV treatment after TBI reduced synaptophysin +  and PSD 95+ puncta decline in the dentate molecular layer at 84  days post-TBI. **(A–T)** Illustrate examples of synaptophysin (Syn) and postsynaptic density 95 protein (PSD95) puncta in the dentate molecular layer between naïve **(A,F,K,P)**, TBI **(B,G,L,Q)**, and TBI + 6.4 × 10^9^ EV **(C,H,M,R)**, TBI + 12.8 × 10^9^ EV **(D,I,N,S)**, and TBI + 25.6 × 10^9^ EV **(E,J,O,T)** groups. The panels in the fourth column are magnified views of boxed regions from the third column displaying putative synapses (arrows). The bar charts **(U–W)** compare the area fraction (AF) of Syn +  and PSD95+ puncta in the dentate molecular layer between different groups. Scale bar in **A** (1 μm) applies to **A–O**. *p < 0.05; ***p < 0.001; NS, not significant.

**Figure 7:**
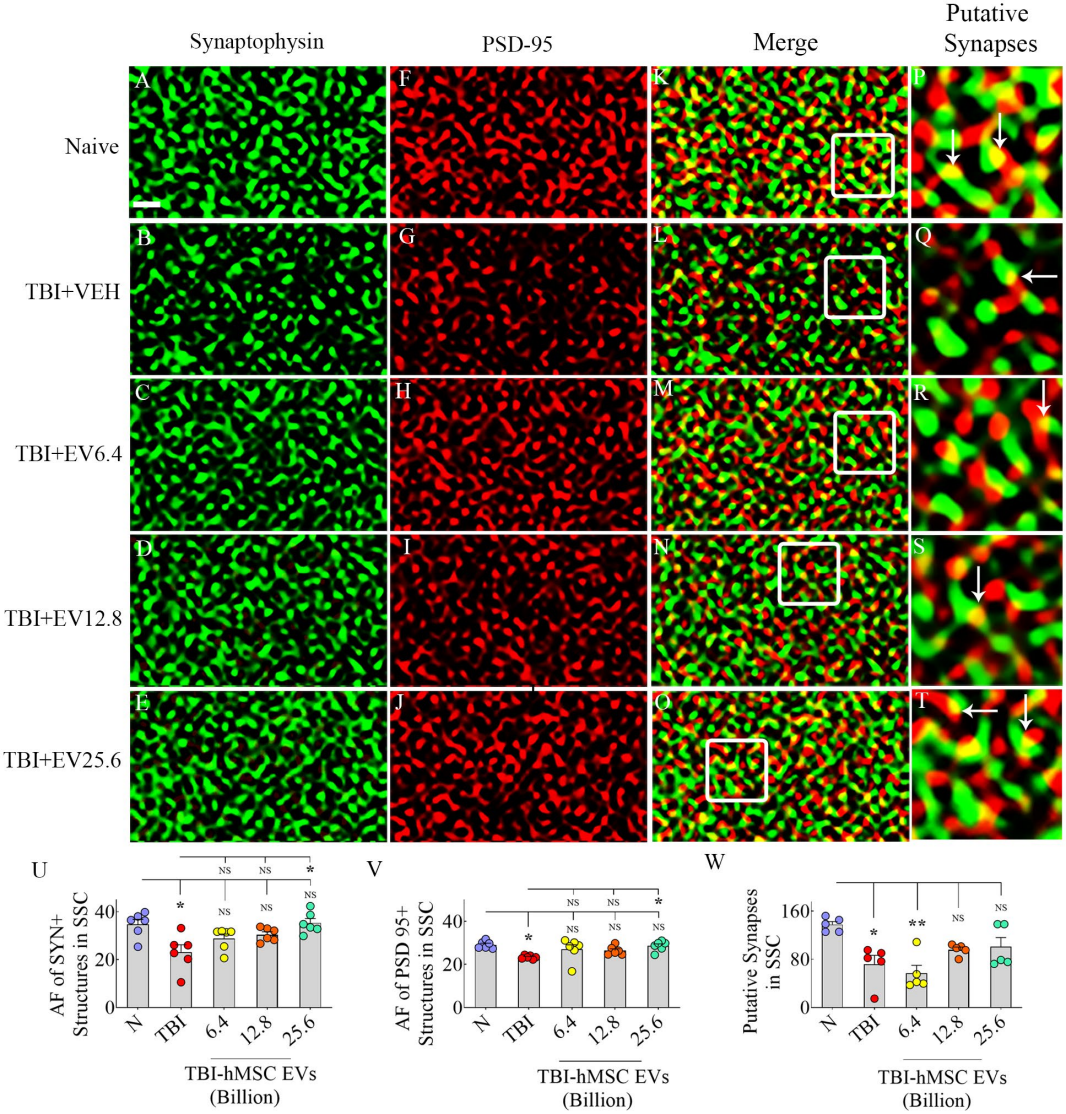
Intranasal hMSC-EV treatment after TBI reduced synaptophysin+ (Syn+) and PSD95+ puncta decline in the somatosensory cortex (SSC) at 84 days post-TBI. **(A–T)** Illustrate examples of Syn and postsynaptic density 95 protein (PSD95) puncta in SSC between naïve **(A,F,K,P)**, TBI **(B,G,L,Q)**, and TBI + 6.4 × 10^9^ EV **(C,H,M,R)**, TBI + 12.8 × 10^9^ EV **(D,I,N,S)**, and TBI + 25.6 × 10^9^ EV **(E,J,O,T)** groups. The panels in the fourth column are magnified views of boxed regions from the third column displaying putative synapses (arrows). The bar charts **(U–W)** compare the area fraction (AF) of Syn +  and PSD95+ puncta in SSC between different groups. Scale bar in **A** (1 μm) applies to **A–O**. *p < 0.05; **p < 0.01; NS, not significant.

We evaluated Syn+ and PSD95+ puncta in the molecular layer of the dentate gyrus and SSC, ipsilateral to CCI. We included SSC in Syn+ and PSD95+ puncta measurements to ascertain potential changes in the synaptic density in the peri-injury area. Representative images of Syn+ and PSD95+ puncta from the dentate molecular layer and SSC, along with potential synapses, are illustrated (Figures 6A–T, 7A–T). ANOVA analysis of area fractions of Syn+ and PSD95+ puncta and putative synapses in both regions revealed significant differences between groups (*p* < 0.05–0.01, Figures 6U–W, 7U–W). The area fractions of Syn+ puncta were reduced in the TBI group compared to the naïve control group in both regions (*p* < 0.05, Figures 6U, 7U). In contrast, TBI + 6.4, 12.8 or 25.6 × 10^9^ EV groups, Syn+ puncta were equivalent to the naïve control group (*p* > 0.05, Figures 6U, 7U). The TBI + 25.6 × 10^9^ EV group also displayed significantly higher Syn+ puncta than the TBI group in both regions (*p* < 0.05, Figures 6U, 7U).

Consistent with the above results, ANOVA analysis of area fractions of PSD95+ puncta and putative synapses in both the molecular layer of the dentate gyrus and SSC revealed significant differences between groups (*p* < 0.05–0.01, Figures 6V,W, 7V,W). In the dentate molecular layer, *post hoc* tests revealed no differences in the density of PSD95+ puncta or putative synapses between the naïve group and different TBI groups (*p* > 0.05, Figures 6V,W). However, the TBI + 25.6 × 10^9^ EV group displayed a higher density of PSD95+ puncta and putative synapses than the TBI group (*p* < 0.05–0.001, Figures 6V,W).

In contrast, in SSC, the area fractions of PSD95+ puncta and putative synapses were reduced in the TBI group compared to the naïve control group (*p* < 0.05, Figures 7V,W). However, in TBI + 6.4, 12.8, or 25.6 × 10^9^ EV groups, PSD95+ puncta were equivalent to the naïve control group (*p* > 0.05, Figure 7V). The TBI + 25.6 × 10^9^ EV group also displayed significantly higher PSD95+ puncta than the TBI group (*p* < 0.05, Figure 6V).

Moreover, in TBI + 12.8 or 25.6 × 10^9^ EV groups, putative synapses were equivalent to the naïve control group (*p* > 0.05, Figure 7W). Thus, hMSC-EV treatment after TBI eased the loss of synapses in both the dentate molecular layer and SSC.

**Figure 8:**
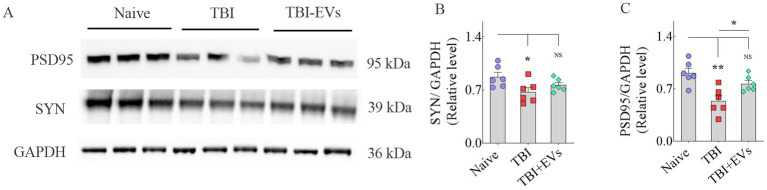
Western blot analysis of synaptophysin + (Syn+) and postsynaptic density protein 95+ (PSD95+) proteins in the somatosensory cortex (SSC) at 84 days post-TBI. The bands in **A** illustrate western blots of PSD95, Syn, and GAPDH proteins from naïve, TBI, and TBI + 25.6 × 10^9^ EV groups. The bar charts **(B,C)** compare the density of Syn +  and PSD95+ proteins in the SSC. *p < 0.05; **p < 0.01; NS, not significant.

Furthermore, western blot analysis of SSC lysates confirmed a significant reduction in both Syn and PSD95 protein levels in TBI mice receiving vehicle compared to naive control mice (*p* < 0.05–0.01, Figures 8A–C). However, TBI mice receiving hMSC-EVs displayed Syn and PSD95 levels closer to naive control mice (*p* > 0.05, Figures 8A–C). Thus, western blot results validate the findings of Syn+ and PSD95+ puncta measurements from the SSC.

## Discussion

The results of this study demonstrate that IN administration of a single optimal dose of hMSC-EVs at 90 min after TBI can ease the TBI-induced decline in hippocampal neurogenesis and putative synapses in the chronic phase of TBI. Sustained maintenance of an optimal level of neurogenesis in TBI mice after hMSC-EV treatment was apparent from both net neurogenesis measured through BrdU and NeuN double labeling and the status of neurogenesis quantified via DCX+ neurons, matching the extent of neurogenesis in the age-matched naïve control mice. Such finding contrasts with vehicle treated TBI mice displaying reduced neurogenesis compared to naïve control mice. Similarly, Syn+ and PSD95+ puncta and protein measurements suggested the loss of synapses in vehicle-treated TBI mice but not in TBI mice receiving hMSC-EVs.

How does hMSC-EV treatment in the acute phase of TBI prevent neurogenesis decline in the chronic phase of TBI? The hMSC-EVs likely mediate these effects by ameliorating chronic neuroinflammation and restraining the decline in BDNF-ERK-CREB signaling after TBI. Neurogenesis is sensitive to chronic neuroinflammation (Ekdahl et al., 2003; Fuster-Matanzo et al., 2013; Gemma and Bachstetter, 2013; Fan and Pang, 2017; Sung et al., 2020), and neuroinflammation is one of the most conspicuous pathological changes seen weeks and months after TBI (Kodali et al., 2023). The sensitivity of neurogenesis to neuroinflammation could be gleaned from reduced hippocampal neurogenesis and cognitive processes across the lifespan after significant neuroinflammation in the developing brain (Green and Nolan, 2014). Microglia play a significant role in neuroinflammation by activating and releasing proinflammatory cytokines such as interleukin-1 beta (IL-1β), IL-6, and tumor necrosis factor-alpha (TNF-α). Furthermore, the regulation of neurogenesis by microglia has been well-documented (Xiong et al., 2016). For example, proinflammatory microglia secreting IL-1β, IL-6, IL-18, TNF-α, and interferon-gamma can diminish neurogenesis by inhibiting NSC proliferation and/or negatively impacting the recruitment of new neurons into behaviorally vital hippocampal networks (Belarbi et al., 2012; Fuster-Matanzo et al., 2013; Gemma and Bachstetter, 2013; Ryan and Nolan, 2016). Moreover, persistent microglial activation interferes with brain tissue repair and promotes neurodegeneration (Lull and Block, 2010; Gyengesi et al., 2019). In contrast, anti-inflammatory microglia secreting IL-4 and IL-10 and transforming growth factor-beta increase neurogenesis (Fuster-Matanzo et al., 2013; Borsini et al., 2020; Zhang J et al., 2021). In this context, TBI leading to a chronic neuroinflammatory condition characterized by nucleotide-binding domain leucine-rich repeat and pyrin domain-containing receptor 3 (NLRP3) inflammasome activation in microglia is relevant (O’Brien et al., 2020). Such activation leads to the overactivation of p38 mitogen-activated protein kinase (p38/MAPK) signaling, causing a constant release of various proinflammatory cytokines in the injured brain (Bachstetter et al., 2013; O’Brien et al., 2020; Kodali et al., 2023). A previous study has shown that IN administration of a single optimal dose of hMSC-EV treatment inhibits NLRP3 inflammasome activation within microglia in the acute phase of TBI (Kodali et al., 2023). Such inflammasome inhibition persisted in the chronic phase of TBI, which prevented the chronic activation of the p38/MAPK signaling pathway resulting in a decreased release of proinflammatory cytokines. Given the above findings, reduced chronic neuroinflammation seen after hMSC-EV treatment has likely contributed to the normalized level of neurogenesis seen in TBI mice receiving hMSC-EVs.

Another potential reason for TBI mice receiving hMSC-EVs maintaining higher levels of neurogenesis in the chronic phase, compared to vehicle-treated TBI mice, is the positive modulation of BDNF-ERK-CREB signaling by hMSC-EVs. BDNF-ERK-CREB signaling is one of the pathways regulating hippocampal neurogenesis (Jiang et al., 2015; Sabbir and Fernyhough, 2018). BDNF binds to tropomyosin receptor kinase B (TrkB; Minichiello, 2009), which activates many intracellular signaling cascades, including the ERK pathway. On the other hand, p-ERK stimulates the phosphorylation of CREB, leading to BDNF transcription via the binding of p-CREB to the BDNF promoter region (Reichardt, 2006). Studies have shown that the BDNF-ERK-CREB pathway can significantly regulate hippocampal neurogenesis and improve cognition (Giachino et al., 2005; Fan et al., 2016). When considered individually, the role of BDNF in maintaining optimal levels of neurogenesis has been well demonstrated in multiple studies (Choi et al., 2018; Okamoto et al., 2021; Zhang J et al., 2021). Similarly, hippocampal neurogenesis is sensitive to changes in ERK signaling (Yan et al., 2007; Liu et al., 2018; Jiang et al., 2021). The involvement of CREB in adult neurogenesis is apparent from newly added neurons in the hippocampus expressing p-CREB (Bender et al., 2001; Nakagawa et al., 2002a,b). Studies have also shown that activation of the cAMP-p-CREB transcription pathway increases neurogenesis via enhanced NSC proliferation, improved neuronal differentiation of newly born cells, and better survival of newly added neurons in the adult hippocampus, whereas inhibition of the cAMP-p-CREB pathway reduces neurogenesis (Nakagawa et al., 2002a,b; Fujioka et al., 2004). Thus, all components of BDNF-ERK-CREB signaling can considerably influence hippocampal neurogenesis.

The current study showed a significant decline in BDNF-ERK-CREB signaling in the injured hippocampus of vehicle-treated TBI mice compared to that of naïve mice. In contrast, BDNF-ERK-CREB signaling in the injured hippocampus of TBI mice receiving an optimal dose of hMSC-EVs was closer to naïve mice. This finding was apparent from measuring concentrations of BDNF, p-ERK1/2, and p-CREB in the hippocampus, which is consistent with the decreased BDNF mRNA and protein expression observed in the injured hippocampus and/or the cortex at different time points after TBI in many previous studies (Gustafsson et al., 2021). Deficient ERK and CREB activation in the hippocampus was also observed after TBI (Atkins et al., 2009). In the current study, hMSC-EV treatment brought the concentrations of BDNF, p-ERK1/2, and p-CREB in the injured hippocampus closer to naïve control levels. However, p-ERK1/2 and BDNF levels did not differ significantly between TBI and TBI + hMSC-EV groups. These results imply that hMSC-EV treatment after TBI can restrain the decline in BDNF-ERK-CREB signaling. Notably, similar BDNF concentrations observed in the acute phase of TBI between naïve mice and TBI mice receiving hMSC-EVs were sustained in the chronic phase of TBI. Such a finding raises a question of how hMSC-EV treatment after TBI can improve BDNF-ERK-CREB signaling in the injured hippocampus.

Improved BDNF-ERK-CREB signaling is likely an indirect effect of lessening chronic neuroinflammation by hMSC-EVs because decreased BDNF concentrations in neurodegenerative diseases have been linked to a chronic neuroinflammatory environment in the brain. Indeed, neuroinflammation can impact several BDNF-related signaling pathways, as proinflammatory signaling cascades lead to neuronal malfunction (Lima Giacobbo et al., 2019). Studies have also shown that increased proinflammatory signaling reduces the BDNF mRNA and protein expression (Guan and Fang, 2006; Gibney et al., 2013). Specifically, activated microglia can regulate BDNF function by lessening BDNF expression and/or its high-affinity receptor TrkB (Frühauf-Perez et al., 2018; Jin et al., 2019). Inflammatory cytokines can also impact the phosphorylation of the BDNF receptor (TrkB), leading to reduced BDNF signaling (Cortese et al., 2011). Furthermore, the proinflammatory cytokine IL-1β can downregulate BDNF in the hippocampus (Tong et al., 2012; Lynch, 2015). Thus, improved BDNF-ERK-CREB signaling after hMSC-EV treatment is at least partially linked to the amelioration of neuroinflammation. Another issue to consider is the role of hMSC-EV cargo. However, none of the top 20 highly enriched miRNAs found within hMSCs are known to upregulate BDNF expression (Kodali et al., 2023). Instead, several miRNAs enriched in hMSC-EVs can decrease BDNF concentration, which includes miR-26b (Caputo et al., 2011), miR-10a-5p (Zhang Q et al., 2021), miR-10b-5p (Müller, 2014), and miR-30a-3p (Mellios et al., 2008). Still, the combined effects of the miRNA and protein cargo of hMSC-EVs improving BDNF-ERK-CREB signaling after TBI cannot be ruled out. To comprehend this issue, additional studies on the proteome of hMSC-EVs are needed.

While most studies on TBI-related pathophysiological changes have been focused on neurodegeneration and neuroinflammation, the role of lost synapses in developing enduring cognitive and mood dysfunction is recognized (Jamjoom et al., 2021). In the CCI model, many studies have suggested reductions in putative synapses in the hippocampus. One study has reported a 66% reduction in synapses in the CA1 subfield at 2 days post-injury, which was spontaneously improved to just a 14% reduction at 30 days post-injury (Scheff et al., 2005). Other studies have suggested a 31% reduction in Syn+ puncta in the dentate gyrus at 3 days post-injury (Gao et al., 2011), reduced PSD95 protein in the hippocampus at 1–7 days post-injury (Wakade et al., 2010), and 27% reduction in VGlut1+ puncta at 1–7 days post-injury (Nikolakopoulou et al., 2016). Such synapse loss has been attributed to increased oxidative stress and neuroinflammation after TBI (Ansari et al., 2008a,b). For example, increased TNF-α concentration after TBI can reduce the expression of α-Amino-3-hydroxy-5-methyl-4-isoxazolepropionic acid (AMPA) glutamate receptor 2 (GluR2) subunits (Stellwagen et al., 2005; Beattie et al., 2010). Furthermore, microglial complement receptor 3 (CR3) activation can trigger longstanding synaptic transmission decline by stimulating NADPH oxidase and internalizing GluR2-containing AMPA receptors (Zhang et al., 2014). Additionally, activated microglia in chronic neuroinflammatory conditions can reduce synapses by engulfing and eliminating presynaptic boutons (Hong et al., 2016; Jamjoom et al., 2021). Thus, TBI can lead to significant synapse loss. Some studies have suggested partial synapse recovery after TBI through neosynaptogenesis (Scheff et al., 2005), but some of this recovery has been suggested to be maladaptive (Huntley, 2012). The current study, through quantification of Syn+ and PSD95+ puncta, showed significantly reduced densities of Syn+ puncta in both the dentate molecular layer and SSC at 84 days post-TBI. However, reductions in PSD95+ puncta and putative synapses were significant only in the SSC. Interestingly, IN administration of hMSC-EVs after TBI prevented the loss of Syn+ puncta in the dentate molecular layer and SSC and the loss of putative synapses in SSC. Western blot studies further validated the findings of Syn+ and PSD95+ puncta measurements from SSC. Considering the role of neuroinflammation and activated microglia in reducing synapses, restrained synapse loss with hMSC-EV treatment after TBI appeared to be linked to the amelioration of neuroinflammation.

## Conclusion

This study has shown that a single IN treatment of an appropriate dose of hMSC-EVs in the acute phase of TBI can ease hippocampal neurogenesis decline and synapse loss in the chronic phase of TBI. Thus, better cognitive and mood function observed in hMSC-EV-treated TBI mice compared to vehicle-treated TBI mice reported in the recently published study (Kodali et al., 2023) is linked to not only the amelioration of chronic neuroinflammation but also the protection of hippocampal neurogenesis and synapses.

## Data availability statement

The original contributions presented in the study are included in the article/supplementary material, further inquiries can be directed to the corresponding author.

## Ethics statement

The animal study was reviewed and approved by the Animal Care and Use Committee of Texas A&M University.

## Author contributions

AKS: concept. AKS, MK, LM, RR, and BM: research design. MK, LM, RR, BM, RU, SA, BS, GS, and AKS: data collection, analysis, and interpretation. MK and AKS: preparation of figure composites and manuscript writing. All authors provided feedback, edits, and additions to the manuscript text and approved the final version.

## Funding

This study was supported by National Institute of Neurological Disorders and Stroke a grant from (1R01NS106907 to AKS).

## Conflict of interest

The authors declare that the research was conducted in the absence of any commercial or financial relationships that could be construed as a potential conflict of interest.

## Publisher’s note

All claims expressed in this article are solely those of the authors and do not necessarily represent those of their affiliated organizations, or those of the publisher, the editors and the reviewers. Any product that may be evaluated in this article, or claim that may be made by its manufacturer, is not guaranteed or endorsed by the publisher.
